# A Mathematical Approach with Fractional Calculus for the Modelling of Children's Physical Development

**DOI:** 10.1155/2019/3081264

**Published:** 2019-09-12

**Authors:** Nisa Özge Önal, Kamil Karaçuha, Göksu Hazar Erdinç, Banu Bahar Karaçuha, Ertuğrul Karaçuha

**Affiliations:** ^1^Informatics Institute, Istanbul Technical University, Istanbul 34467, Turkey; ^2^Medicine Faculty, Istanbul Medeniyet University, Istanbul 34000, Turkey

## Abstract

From birth to now, it is getting more and more important to keep track of the children development, because knowing and determining the factors related to the physical development of the children would provide better and reliable results for children care. In this study, we developed a mathematical approach to have the ability of analysing and examining factors such as weight, height, and body mass index with respect to the age. We used 7 groups for weight, height, and body mass index in Percentage Chart of Turkey. We developed a continuous curve which is valid for any time interval by using discrete weight, height, and body mass index data of 0–18 years old children and the least squares method. By doing so, it became possible to find the percentage and location of the children in Percentage Chart. We advanced a new mathematical model with the help of fractional calculus theory. The results are quite successful and better compared to linear and Polynomial Model analysis. The method provides the opportunity to predict expected values of the children for the future by using previous data obtained in the development of the children.

## 1. Introduction

Fractional integral and derivative, are distinctly defined as the derivative and integral with non-integer order. The concept of fractional calculus, which means in more general form, the calculus of integrals and derivatives of any arbitrary real or complex order, is built up from a question raised in 1695 by French Mathematician Marquis de L'Hôpital (1661–1704) to Wilhelm Leibniz (1646–1716) [1–3]. He asked what if derivative order becomes 0.5, and Leibniz's response to this question was “this is an apparent paradox from which, one day, useful consequences will be drawn …” [1–6].

For 50 years, many mathematicians, engineers, scientists, and researchers prove in their studies that, fractional derivatives and integrals contain important information about the systems that they are searching for. Especially, the fractional derivative provides pretty good insight for the memory and hereditary of a process or phenomena. The fractional calculation is widely used in the control theory, mechanics, and economics, finance, electromagnetic and mostly in biology [7–18]. In this study, we advanced a mathematical approach which analyses and examines the factors continuously related to the physical development of the children by the help of fractional calculus theory. We used 7 groups for body weight, height, and body mass index in Percentage Chart of Turkey. We developed a continuous curve valid for any time interval by using discrete weight, height, and body index data of 0–18 years old children and least squares method. Therefore, it became possible to find the percentage and location of the children in Percentage Chart. The results of the fractional calculus model analysis are more successful than the linear and Polynomial Model analysis. The method provides the opportunity to predict expected values of the children for the future by using previous data obtained in the development of the children. In this paper, the theory, numerical results of the developed theory and comparison with the other modelling methods such as linear and polynomial methods are presented.

## 2. Formulation of the Problem

Firstly, fractional derivative *𝒟*_*x*_^*α*^ determined from the Riemann-Liouville equation [5] which has the form;

(1) $$\begin{array}{c} D_{x}^{\alpha }f\left(x\right)=\frac{d^{\alpha }f\left(x\right)}{dx^{\alpha }}=\frac{1}{\Gamma \left(1-\alpha \right)}\frac{d}{dx}\int_{-\infty }^{x}\frac{f\left(t\right)}{{\left(x-t\right)}^{\alpha }}dt. \end{array}$$

Here, Γ(1 − *α*) is a Gamma Function which is defined as  Γ(1 − *α*)=∫_0_^*∞*^*t*^−*α*^*e*^−*t*^*dt*. The Fractional Order (FO), *α* varies from 0 to 1.

In order to improve the convergence of the Polynomial Model results, we utilized the theory of fractional calculus [4, 5]. The vital question we asked in this paper is what if the fractional derivative of *f*(*x*) is equal to the expression given in equation (2). Derivative order is *α* and *αε* (0, 1).

(2) $$\begin{array}{c} D_{x}^{\alpha }f\left(x\right)=\frac{d^{\alpha }f\left(x\right)}{dx^{\alpha }}=\overset{\infty }{\underset{n=1}{\sum }}a_{n}nx^{n-1}. \end{array}$$

Here, *f*(*x*) corresponds to the data of children's weight, height, and body mass index with respect time which is denoted as *x* in equation (2).

After, Laplace transform of equation (2) is taken [4].

(3) $$\begin{array}{c} ℒ\left\{f\left(x\right)\right\}=F\left(s\right)=\frac{f\left(0\right)}{s}+\overset{\infty }{\underset{n=1}{\sum }}\frac{a_{n}}{s^{\alpha +n}}\Gamma \left(n+1\right), \end{array}$$

*ℒ* stands for the Laplace Transform and *ℒ*^−1^ stands for the inverse Laplace transform. Laplace transform of *f*(*x*) is denoted as *F*(*s*). Inverse Laplace transform of equation (3) is given as

(4) $$\begin{array}{c} {ℒ}^{-1}\left\{F\left(s\right)\right\}=f\left(x\right)=f\left(0\right)+\overset{\infty }{\underset{n=1}{\sum }}\frac{a_{n}\Gamma \left(n+1\right)x^{\alpha +n-1}}{\Gamma \left(\alpha +n\right)}\;. \end{array}$$

As mentioned in the introduction part, our purpose is to model the children's physical development with respect to time by using previously found data, and we use the least square mean method to achieve our goal [19, 20]. Due to having the finite number of discrete data, summation corresponds to  *f*(*x*) in equation (3) also needs to be truncated to *N*. Truncated version of equation (4) is given in

(5) $$\begin{array}{c} f\left(x\right)≅f\left(0\right)+\overset{N}{\underset{n=1}{\sum }}\frac{a_{n}\Gamma \left(n+1\right)x^{\alpha +n-1}}{\Gamma \left(\alpha +n\right)}. \end{array}$$

We have a dataset to make regression on it.

(6) $$\begin{array}{c} P_{K}=\left[p_{0}p_{1}\ldots p_{K}\right],\\ x_{K}=\left[x_{0}x_{1}\ldots x_{K}\right]. \end{array}$$

The dimension of the dataset is *N*+1. In the dataset, the corresponding value for each *x*_*i*_ is given as *P*_*i*_. Here, *x*_*i*_ represents the time, and *P*_*i*_ corresponds to the weight, height, and body mass index of the child in the specific time. The dimension of the dataset determines the upper limit of *N* value given in equation (5) by the nature of solving the System of Linear Algebraic equations (SLAE) [19].

The error between the value *p*_*i*_ and *f*(*x*_*i*_) is showed as *ε*_*i*_ in equation (7). In the least squares method, the purpose is to minimize the square of the total error contributing from each data points.

(7) $$\begin{array}{c} {\left(\varepsilon_{i}\right)}^{2}={\left(p_{i}-f\left(x_{i}\right)\right)}^{2}. \end{array}$$

In equation (8), summation of error's square is given.

(8) $$\begin{array}{c} \varepsilon_{T}^{2}={\overset{K}{\underset{i=0}{\sum }}\left[p_{i}-\left\{f\left(0\right)+\overset{N}{\underset{n=1}{\sum }}\frac{a_{n}\Gamma \left(n+1\right)x_{i}^{\alpha +n-1}}{\Gamma \left(\alpha +n\right)}\right\}\right]}^{2}. \end{array}$$

In order to minimize the total error given in equations (8) and (9) needs to be satisfied [19].

(9) $$\begin{array}{c} \frac{\partial \varepsilon_{T}^{2}}{\partial f\left(0\right)}=0,\frac{\partial \varepsilon_{T}^{2}}{\partial a_{1}}=0,\frac{\partial \varepsilon_{T}^{2}}{\partial a_{2}}=0,\ldots ,\frac{\partial \varepsilon_{T}^{2}}{\partial a_{K}}=0. \end{array}$$

After finding equations (8) and (9), following SLAE is achieved. SLAE can be denoted as

(10) $$\begin{array}{c} {\left[A\right]}_{N+1\times N+1}{\left[\Omega \right]}_{N+1\times 1}={\left[B\right]}_{N+1\times 1}, \end{array}$$

where,

(11) $$\begin{array}{c} A=\left[\begin{array}{ccccc} k+1 & \frac{1}{\Gamma \left(\alpha +1\right)}\overset{K}{\underset{i=0}{\sum }}x_{i}^{\alpha } & \frac{2.1!}{\Gamma \left(\alpha +2\right)}\overset{K}{\underset{i=0}{\sum }}x_{i}^{\alpha +1} & \ldots & \frac{n!}{\Gamma \left(\alpha +n\right)}\overset{K}{\underset{i=0}{\sum }}x_{i}^{\alpha +n-1}\\ \overset{K}{\underset{i=0}{\sum }}x_{i}^{\alpha } & \frac{1}{\Gamma \left(\alpha +1\right)}\overset{K}{\underset{i=0}{\sum }}x_{i}^{2\alpha } & \frac{2.1!}{\Gamma \left(\alpha +2\right)}\overset{K}{\underset{i=0}{\sum }}x_{i}^{2\alpha +1} & \ldots & \frac{n!}{\Gamma \left(\alpha +n\right)}\overset{K}{\underset{i=0}{\sum }}x_{i}^{2\alpha +n-1}\\ \overset{K}{\underset{i=0}{\sum }}x_{i}^{\alpha +1} & \frac{1}{\Gamma \left(\alpha +1\right)}\overset{K}{\underset{i=0}{\sum }}x_{i}^{2\alpha +1} & \frac{2.1!}{\Gamma \left(\alpha +2\right)}\overset{K}{\underset{i=0}{\sum }}x_{i}^{2\alpha +2} & \ldots & \frac{n!}{\Gamma \left(\alpha +n\right)}\overset{K}{\underset{i=0}{\sum }}x_{i}^{2\alpha +n}\\ \vdots & \vdots & \vdots & \vdots & \vdots \\ \overset{K}{\underset{i=0}{\sum }}x_{i}^{\alpha +n-1} & \frac{1}{\Gamma \left(\alpha +1\right)}\overset{K}{\underset{i=0}{\sum }}x_{i}^{2\alpha +n-1} & \frac{2.1!}{\Gamma \left(\alpha +2\right)}\overset{K}{\underset{i=0}{\sum }}x_{i}^{2\alpha +n} & \ldots & \frac{n!}{\Gamma \left(\alpha +n\right)}\overset{K}{\underset{i=0}{\sum }}x_{i}^{2\left(\alpha +n-1\right)} \end{array}\right],\\ \left[\Omega \right]={\left[\begin{array}{ccccc} f\left(0\right) & a_{1} & a_{2} & \ldots & a_{n} \end{array}\right]}^{\text{T}},\\ \left[\text{B}\right]={\left[\begin{array}{ccccc} \overset{K}{\underset{i=0}{\sum }}P_{i} & \overset{K}{\underset{i=0}{\sum }}P_{i}x_{i}^{\alpha } & \overset{K}{\underset{i=0}{\sum }}P_{i}x_{i}^{\alpha +1} & \ldots & \overset{K}{\underset{i=0}{\sum }}P_{i}x_{i}^{\alpha +n-1} \end{array}\right]}^{\text{T}}. \end{array}$$

Here, *T* is the matrix transpose. Unknown coefficients in the vector *Ω* can be found by equation

(12) $$\begin{array}{c} {\left[\Omega \right]}_{N-1\times 1}={\left[A\right]}_{N-1\times N-1}^{-1}{\left[B\right]}_{N-1\times 1}, \end{array}$$

where, [*A*]^−1^ is the inverse of [*A*] matrix.

## 3. Dataset

In this study, we use body weight, height, and body mass index of 0–18 years old children indicated in Percentage Chart of Turkey. The dataset includes 7 groups (3-10-25-50-75-85-97 percentiles for body weight and height, 5-15-25-50-75-85-95 percentiles for body mass index) for boys and girls. See Tables [Supplementary-material supplementary-material-1]–[Supplementary-material supplementary-material-1] in the Supplementary Material [20, 21].

## 4. Results and Discussion

In this study, Polynomial Model and Fractional Model were used for comparing different *N* exponent values up to 3, 4, and 5 (*N*=3,4,5) in equation (5);

We obtained results from Linear, Polynomial, and Fractional Model respectively. Mean Absolute Percentage Error (MAPE) was used for comparing the models [19]. MAPE formulation is showed in equation (13). Tables 1–3 illustrates the results of the age versus body weight, age versus height, and age versus body mass index, respectively.

(13) $$\begin{array}{c} \text{MAPE}=\frac{1}{k}\overset{k}{\underset{i=1}{\sum }}\left|\frac{v\left(i\right)-\tilde{v}\left(i\right)}{v\left(i\right)}\right|\times 100, \end{array}$$

where *v*(*i*) is real value and $\tilde{v}\left(i\right)$ is predicted value.

When fractional order (*α*) value in the Fractional Model is equal to one, Polynomial and Fractional Model are equal to each other as mathematically. In the Fractional Model, alpha values were taken between the 0.001 and 1 increase by 0.001. So, some results can be the same both in Polynomial Model and Fractional Model. Alpha values were determined according to minimum MAPE values.

Table 1 shows MAPE results of the age versus height according to methods of Linear, Polynomial, and Fractional Model. The best results were obtained from Fractional Model in the age versus height data. Considering Table 1, when the truncation number *N* in equation (5) was increased, MAPE ratio in both Polynomial and Fractional Models were decreased as expected.

Average of the total MAPE (AMAPE) was calculated with the formula given in

(14) $$\begin{array}{c} \text{AMAPE}=\frac{{\sum \text{MAPE}}^{}}{M}, \end{array}$$

where, *M* represents the number of values, which was 14.

In order to compare the Polynomial Model and Fractional Model, ratio of MAPE results of each model were calculated. We divided each Polynomial Model's MAPE value by the corresponding Fractional Model's MAPE value. This transaction was applied for all the *N* values where *N* is equal to 3, 4, and 5 respectively. In addition to this, after doing each calculation, the maximum and the minimum values were chosen as the limits. According to the limits, MAPE results evaluated by Polynomial Model was at least 2.01 times and at most 3.95 times greater than MAPE results evaluated by Fractional Model.

By applying equation (5), for *N*=3, AMAPE was 3.11 in the Polynomial Model, whereas AMAPE was found as 1.26 in the Fractional Model. Approximately, AMAPE was found by the Polynomial Model was 2.5 times greater than AMAPE was found by Fractional Model. When *N* was equal to 4 and 5, AMAPE values were 1.58 and 1.18 in the Polynomial Model, whereas AMAPE values were found as 0.49 and 0.43 in the Fractional Model, respectively. Their approximate ratios in order were 3.22 and 2.74.

Table 2 indicates MAPE results of age versus body weight according to methods of Linear, Polynomial, and Fractional Model. According to these results, the most successful model is the Fractional Model, because we obtained the best results for all age versus height data from the Fractional Model.

For Table 2, we can indicate that when the truncation number *N* in equation (5) was increased, MAPE ratio in both polynomial and Fractional Models were decreased. When we applied truncation mentioned in equation (5) (for calculating MAPE results), MAPE results we got from Polynomial Model was, at least 1.18 times and at most 3.65 times greater than the MAPE results we got from Fractional Model. For *N*=3, AMAPE was 11.22 is found by the Polynomial Model, whereas, in the Fractional Model, AMAPE was found as 5.77. Approximately, AMAPE was found by the Polynomial Model was 1.95 times greater than AMAPE was found by the Fractional Model. When *N* was equal to 4 and 5, AMAPE values were 3.88 and 3.48 in the Polynomial Model, whereas AMAPE values were found as 2.2 and 1.87 in the Fractional Model, respectively. Their approximate ratios in order were 1.76 and 1.86.

Table 3 illustrates MAPE results of the age versus body mass index according to methods of Linear, Polynomial, and Fractional Model.

For Table 3, when the truncation number *N* in equation (5) was increased, MAPE ratio in both Polynomial and Fractional Model were decreased. MAPE results we got from Polynomial Model was, at least 0.99 (or 1) times and at most 5.1 times greater than the MAPE results we got from Fractional Model. For *N*=3, AMAPE was 2.81 was found by the Polynomial Model, whereas, in the Fractional Model, AMAPE was found as 2.18. Approximately, AMAPE was found by the Polynomial Model was 1.3 times greater than AMAPE was found by the Fractional Model. When *N* was equal to 4 and 5, AMAPE values were 2.4 and 2.15 in the Polynomial Model, whereas AMAPE values were found as 0.8 and 0.78 in the Fractional Model, respectively.

**Figure 1:**
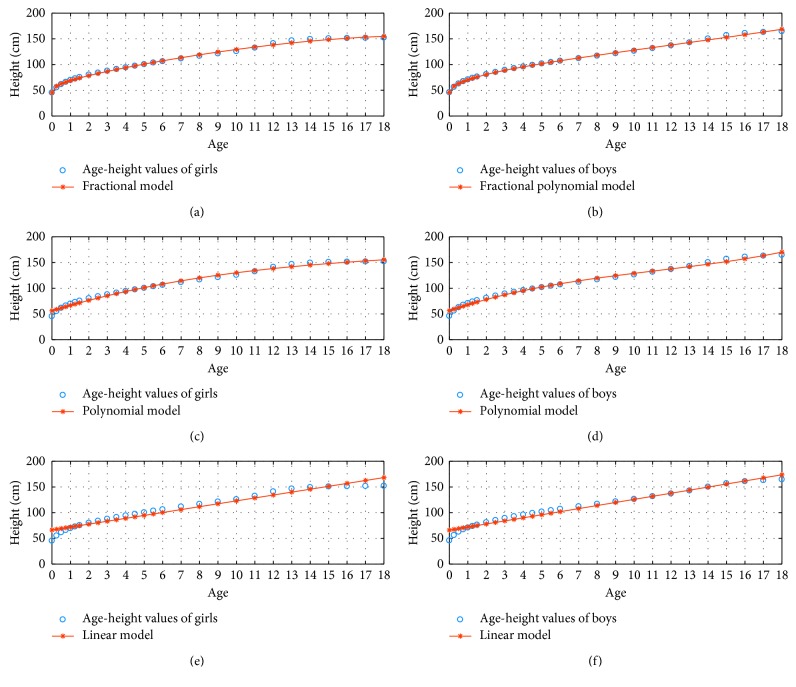
3 percentile age-height boy and girl for *N*=3.

Their approximate ratios in order were 2.89 and 2.76. The Fractional Model is more successful than the Polynomial Model as seen in the results of Tables 1–3. According to these results, we achieved better results with less computational cost using the Fractional Model. In other words, in order to have same MAPE for both methods, the Fractional Method uses less term compared to Polynomial Method.  Figure 1 demonstrates 3 percentile age versus height graphs in the fractional, polynomial, and linear model for *N*=3. Fractional Model has the minimum error for modelling discrete age versus body weight values. A continuous curve valid was developed with the Fractional Model.

**Figure 2:**
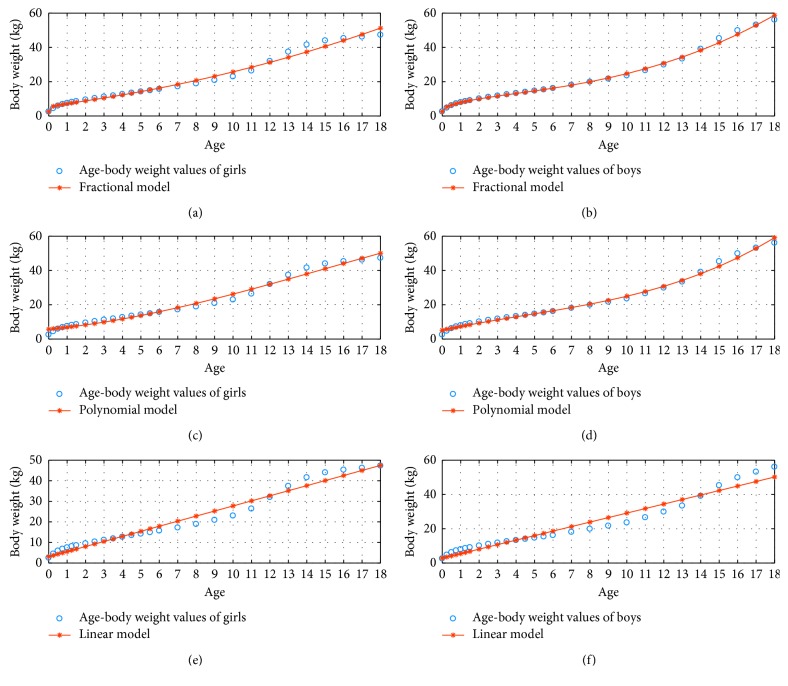
3 percentile age-body weight boy and girl for *N*=3.

Figure 2 represents the 3 percentile age versus body weight graphs using the fractional, polynomial, and linear model for *N*=3.

**Figure 3:**
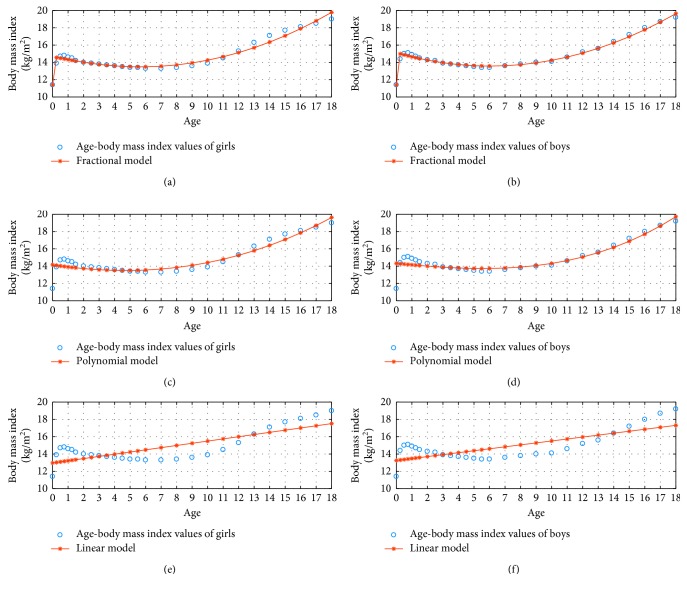
3 percentile age-body mass index boy and girl for *N*=3.

Figure 3 shows the 3 percentile age versus body mass index graphs in the fractional, polynomial, and linear model for *N*=3.

**Figure 4:**
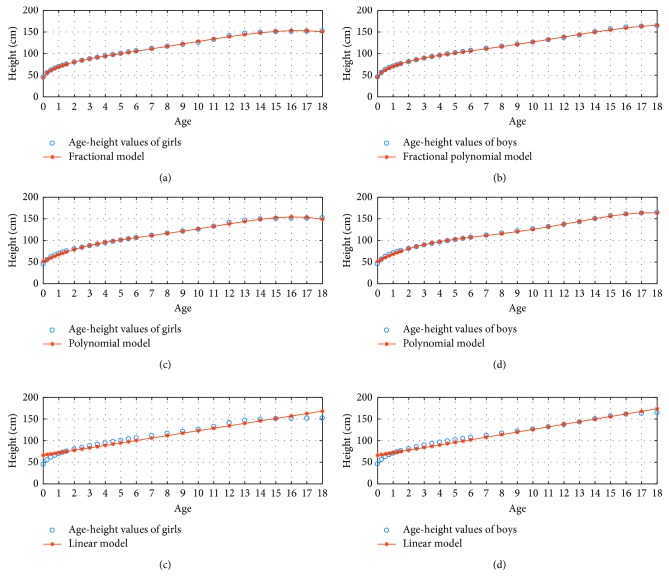
3 percentile age-height boy and girl for *N*=4.

Figure 4 illustrates 3 percentile age versus height graphs using the fractional, polynomial, and linear model for *N*=4.

**Figure 5:**
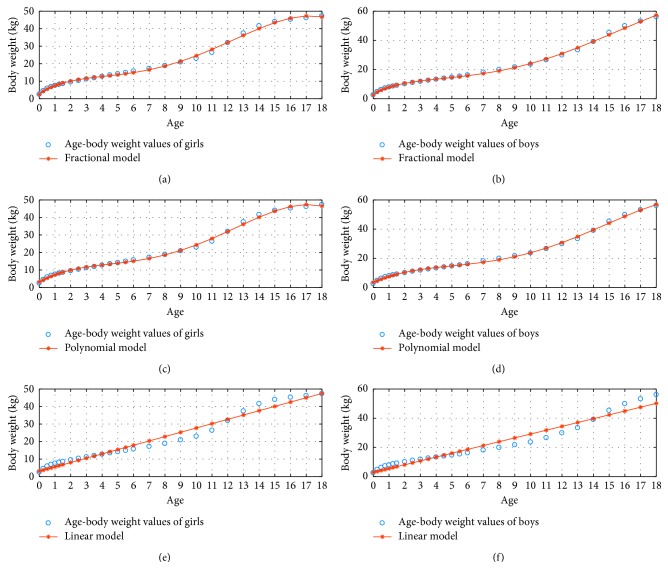
3 percentile age-body weight boy and girl for *N*=4.

Figure 5 demonstrates the 3 percentile age versus body weight graphs using the fractional, polynomial, and linear model for *N*=4.

**Figure 6:**
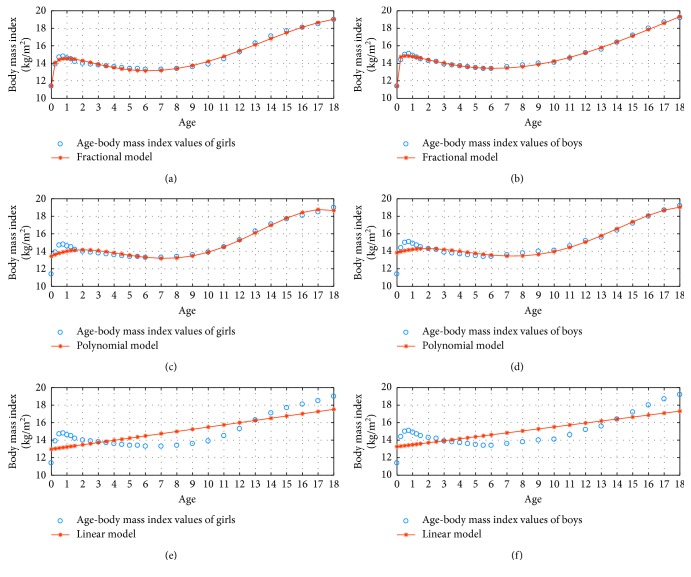
5 percentile age-body mass index boy and girl for *N*=4.

Figure 6 shows the 5 percentile age versus body mass index graphs using the fractional, polynomial, and linear model for *N*=4.

**Figure 7:**
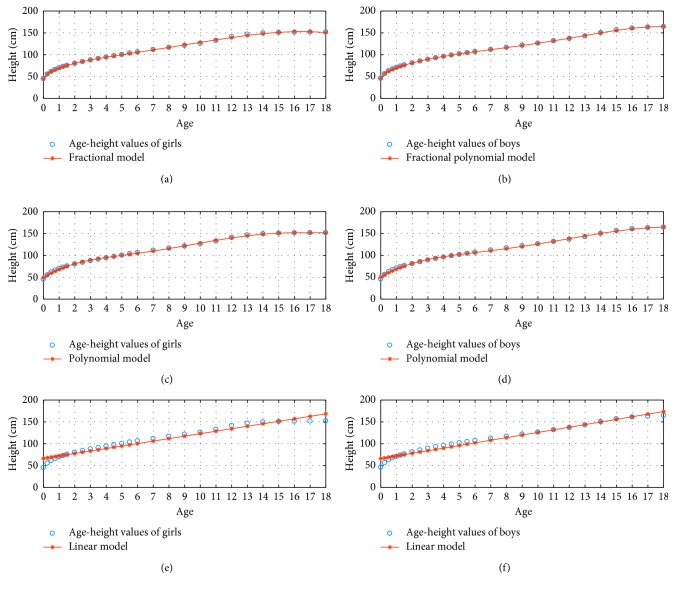
3 percentile age-height boy and girl for *N*=5.

Figure 7 demonstrates the 3 percentile age versus height graphs using the fractional, polynomial, and linear model for *N*=5.

**Figure 8:**
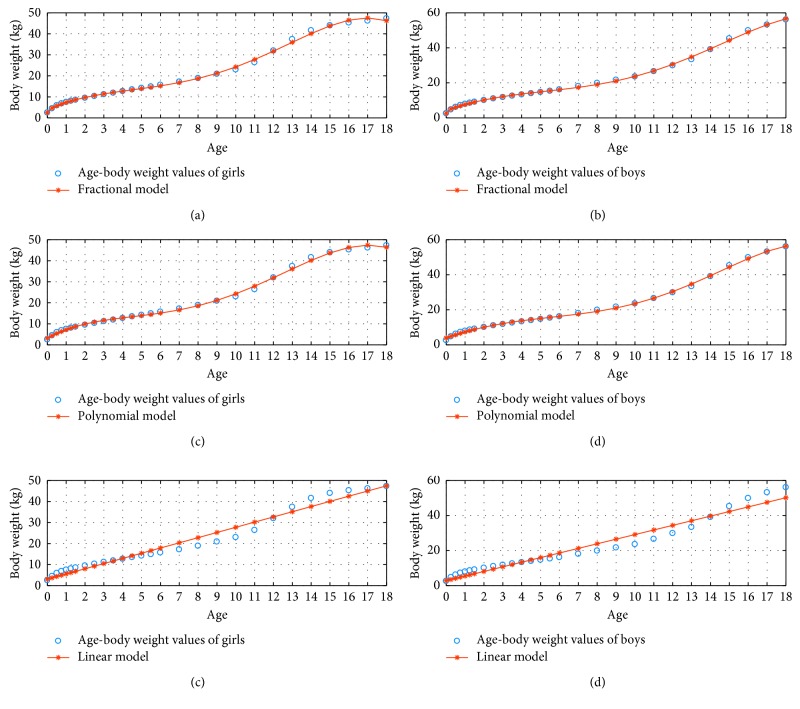
3 percentile age-body weight boy and girl for *N*=5.

Figure 8 represents the 3 percentile age versus body weight graphs in fractional, polynomial, and linear model for *N*=5.

**Figure 9:**
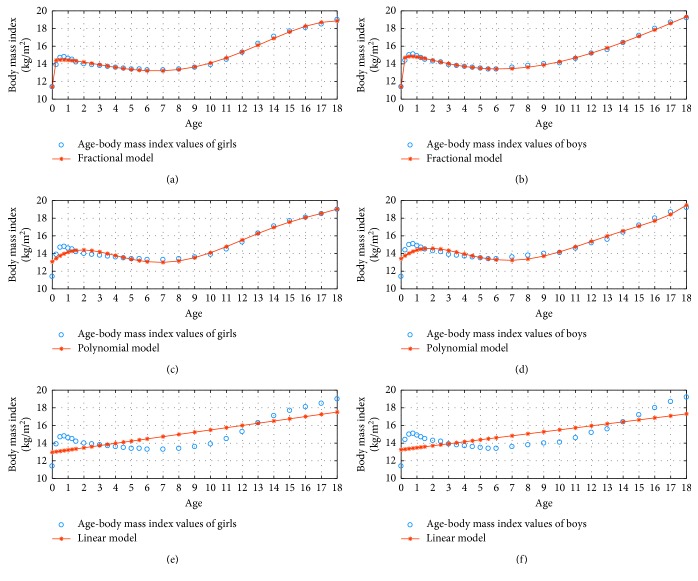
5 percentile age-body mass index boy and girl for *N*=5.

Figure 9 shows the 5 percentile age versus body mass index graphs using the fractional, polynomial, and linear model for *N*=5.

## 5. Conclusions

In this study, we developed a continuous curve valid for any time interval with least squares method by using data related to 7 groups for discrete height, body weight, and body mass index data of 0–18 years old children in Turkey. By doing so, it is possible to find the percentage and location of the child in Percentage Chart. Here, with the help of the fractional calculus theory, a new mathematical model is developed. The results are quite successful and better compared to the linear and Polynomial Model analysis. The method provides the opportunity to predict expected values of the child for the future by using previous data obtained in the development of child process.

The method we suggest is modelling the Age-Body Height, Age-Body Weight, and Age-Body Mass Index by quite less error with respect to the well-known polynomial and linear method. By the suggested method, one can have the children's development parameters in any desired time by having more accurate continuous curve achieved via using fractional method and discrete data for each percentile.

The results of Age versus Body Height in Linear, Polynomial, and Fractional Model from 3 to 5 exponent numbers are showed in Table 1. For Table 1, when the truncation number *N* in equation (5) was increased, MAPE ratio in both polynomial and Fractional Models were decreased. MAPE results evaluated by Polynomial Models were at least 2.01 times and at most 3.95 times greater than MAPE results evaluated by Fractional Models. For *N*=3, AMAPE was 3.11 in the Polynomial Model, and 1.26 in the Fractional Model. Approximately, AMAPE was found by the Polynomial Model was 2.5 times greater than AMAPE was found by the Fractional Model. When *N* was equal to 4 and 5, AMAPE values were 1.58 and 1.18 in the Polynomial Model, whereas AMAPE values were found as 0.49 and 0.43 in the Fractional Model, respectively.

The results of Age-Body Weight in Linear, Polynomial, and Fractional Model from 3 to 5 exponent numbers are illustrated in Table 2. Table 2 consists MAPE results of age-body weight according to Linear, Polynomial, and Fractional Model methods. According to these results, the most successful model is the Fractional Model. We got the best results with the Fractional Model for all age-height data. For Table 2, when the truncation number *N* in equation (5) was increased, MAPE ratio in both polynomial and Fractional Models were decreased. MAPE results evaluated by Polynomial Models were, at least 1.18 times and at most 3.65 times greater than the MAPE results evaluated by Fractional Models. For *N*=3, AMAPE was 11.22 by the Polynomial Model, whereas, in the Fractional Model, AMAPE was found as 5.77. Approximately, AMAPE was found by the Polynomial Model was 1.95 times greater than AMAPE was found by the Fractional Model. When *N* was equal to 4 and 5, AMAPE values were 3.88 and 3.48 in the Polynomial Model, whereas AMAPE values were found as 2.2 and 1.87 in the Fractional Model, respectively.

The MAPE results of age versus body mass index considering Linear, Polynomial, and Fractional Model are showed in Table 3. When the truncation number *N* in equation (5) was increased, MAPE ratio in both polynomial and Fractional Models were decreased. MAPE results evaluated by Polynomial Models were, at least 1 times and at most 5.1 times greater than the MAPE results evaluated by Fractional Models. For *N*=3, AMAPE was 2.81 was found by the Polynomial Model, whereas, in the Fractional Model, AMAPE was found as 2.18. Approximately, AMAPE was found by the Polynomial Model was 1.3 times greater than AMAPE was found by the Fractional Model. When *N* was equal to 4 and 5, AMAPE values were 2.4 and 2.15 in the Polynomial Model, whereas AMAPE values were found as 0.83 and 0.78 in the Fractional Model, respectively.

## Figures and Tables

**Table 1:** results of age-body height to linear, Polynomial, and Fractional Model from 3 to 5 exponent numbers.

Results	Linear model	*N*=3			*N*=4			*N*=5		
Age-height		Polynomial model	Fractional model		Polynomial model	Fractional model		Polynomial model	Fractional model	
	MAPE	MAPE	MAPE	Alfa	MAPE	MAPE	Alfa	MAPE	MAPE	Alfa
3%-boy	5.402163228	2.947443454	0.879511614	0.506	1.450408	0.570475167	0.657	1.387619	0.43146	0.547
10%-boy	5.407534646	3.01494031	0.963707668	0.495	1.413681	0.534720874	0.661	1.337368	0.387715	0.56
25%-boy	5.464773802	3.060094876	1.034929851	0.483	1.40315	0.491194116	0.66	1.317915	0.363516	0.563
50%-boy	5.559953069	3.110350759	1.128587385	0.474	1.373834	0.440018959	0.665	1.272034	0.344825	0.587
75%-boy	5.632404553	3.163640075	1.22741669	0.462	1.391571	0.422563403	0.666	1.246001	0.354468	0.575
90%-boy	5.69813391	3.212386377	1.318764065	0.455	1.408876	0.435351278	0.671	1.224827	0.373018	0.617
97%-boy	5.776556168	3.272317243	1.40963534	0.447	1.422935	0.453567536	0.674	1.230354	0.393108	0.63
3%-girl	6.823285327	3.291558273	1.473083602	0.411	1.763813	0.605919002	0.62	1.344039	0.604952	0.622
10%-girl	6.9437678	3.218607376	1.431390254	0.416	1.736411	0.536266756	0.639	1.249269	0.549924	0.636
25%-girl	7.063383601	3.173419071	1.407654414	0.421	1.725992	0.475034474	0.64	1.149177	0.50914	0.7
50%-girl	7.193639107	3.0837981	1.357629583	0.427	1.719019	0.434119083	0.639	1.056893	0.463723	0.713
75%-girl	7.323473266	3.036228472	1.3501217	0.432	1.745766	0.446222807	0.637	0.983734	0.432988	0.715
90%-girl	7.450645847	2.991379294	1.337844927	0.438	1.749846	0.459484184	0.638	0.924253	0.429191	0.721
97%-girl	7.553575607	2.952629006	1.317973151	0.441	1.779546	0.486929947	0.636	0.882858	0.437864	0.742
AMAPE (*M* = 14)	6.378092137	3.109199477	1.2598750174		1.577489142	0.485133399		1.18616721	0.4339922	

**Table 2:** Results of age-body weight to linear, polynomial, and Fractional Model from 3 to 5 exponent numbers.

Results	Linear model	*N*=3			*N*=4			*N*=5		
Age-body weight		Polynomial model	Fractional model		Polynomial model	Fractional model		Polynomial model	Fractional model	
	MAPE	MAPE	MAPE	Alfa	MAPE	MAPE	Alfa	MAPE	MAPE	Alfa
3%-boy	15.94568859	8.124363119	2.225477998	0.51	3.984755	2.420906859	0.783	4.609962	2.36093	0.326
10%-boy	15.94129044	9.022159279	2.866036088	0.427	3.694552	2.313749619	0.787	3.961816	1.907708	0.376
25%-boy	16.10456514	9.835869013	3.633821445	0.342	3.483822	2.23629996	0.789	3.602703	1.476948	0.413
50%-boy	16.29961128	10.85175892	4.679024283	0.233	3.355553	2.12993533	0.785	3.358665	1.155057	0.465
75%-boy	16.29774355	11.7282567	5.574924231	0.134	3.317515	2.096470767	0.777	3.302717	1.209806	0.588
90%-boy	16.32249564	12.34877076	6.117002651	0.044	3.308995	1.944523119	0.76	3.19587	1.428124	0.043
97%-boy	16.0897	12.71400353	6.630194381	0.001	3.574042	1.792015967	0.733	3.092366	1.584805	0.001
3%-girl	13.26776061	12.7520431	6.308625438	0.161	3.945692	3.274024692	0.872	3.951722	2.24351	0.577
10%-girl	12.49925264	12.56753438	6.428139803	0.091	3.70836	2.868085155	0.841	3.695239	2.096702	0.694
25%-girl	11.66779732	12.25039184	6.319341635	0.001	3.548289	2.512683772	0.81	3.498459	2.094255	0.179
50%-girl	10.9204447	11.89221322	6.476098513	0.001	3.564722	2.080363121	0.762	3.36231	1.920233	0.001
75%-girl	10.08145527	11.55082465	7.025518322	0.001	3.919967	1.717374894	0.714	3.216609	2.127784	0.001
90%-girl	9.559115908	10.95681344	7.742623118	0.001	4.897397	1.589039883	0.641	3.061346	2.341874	0.866
97%-girl	9.216849965	10.54687482	8.867764628	0.001	5.917726	1.887806799	0.567	2.868494	2.171694	0.869
AMAPE (*M* = 14)	13.586697932	11.22441976	5.778185181		3.87295621	2.204519995		3.48416271	1.8656735	

**Table 3:** Results of age-body mass index to linear, polynomial, and Fractional Model from 3 to 5 exponent numbers.

Results	Linear model	*N*=3			*N*=4			*N*=5		
Age-body mass index		Polynomial model	Fractional model		Polynomial model	Fractional model		Polynomial model	Fractional model	
	MAPE	MAPE	MAPE	Alfa	MAPE	MAPE	Alfa	MAPE	MAPE	Alfa
5%-boy	6.865466551	2.705373834	0.911126	0.001	2.63779053	0.651071	0.119	2.588440465	0.648818	0.129
15%-boy	6.915739985	2.704290105	1.319749	0.001	2.574127515	0.501764	0.101	2.425287783	0.499791	0.123
25%-boy	6.971573965	2.751387954	1.687045	0.001	2.515566244	0.558212	0.117	2.312195228	0.556502	0.137
50%-boy	7.016885183	2.810732896	2.213369	0.001	2.490436568	0.673523	0.142	2.14042388	0.652327	0.171
75%-boy	7.119935512	3.003870741	2.850619	0.001	2.570392019	0.87928	0.183	2.141484131	0.889948	0.217
85%-boy	7.114096202	3.018708576	3.018709	1	2.627532458	0.969604	0.205	2.060247302	0.973459	0.244
95%-boy	7.148701543	3.13331193	3.133312	1	2.804215436	1.121725	0.157	2.084685295	1.11742	0.278
5%-girl	6.855258661	3.231028281	1.659999	0.017	2.194516478	1.048393	0.261	2.264588115	0.925621	0.046
15%-girl	6.611929356	3.033530893	1.868429	0.001	2.086613692	0.946344	0.232	2.091314011	0.80253	0.065
25%-girl	6.465961229	2.968299158	2.088156	0.001	2.088687566	0.878275	0.218	2.043429311	0.794113	0.076
50%-girl	6.116695931	2.669341113	2.525415	0.001	2.106124203	0.865623	0.195	1.997292855	0.803468	0.069
75%-girl	5.740206516	2.516997789	2.516998	1	2.19501569	0.845701	0.1	1.957301328	0.85037	0.099
85%-girl	5.425758797	2.423741636	2.423742	1	2.253149389	0.767296	0.099	1.915490246	0.754622	0.139
95%-girl	5.270970139	2.383449508	2.38345	1	2.443491251	0.873101	0.043	2.064601668	0.677318	0.18
AMAPE (*M* = 14)	6.545655683	2.811004601	2.185722		2.399118502	0.827136		2.149055829	0.7818790	

## Data Availability

The data used to support the findings of this study are included within the supplementary information file.

## Supplementary Materials

Supplementary Materials Table S1: age-height percentile values of 0–18 years old children. Table S2: age- body weight percentile values of 0–18 years old children. Table S3: age- body mass index percentile values of 0–18 years old children.
