# Supplementary material for: A Mathematical Approach with Fractional Calculus for the Modelling of Children's Physical Development
Source: Comput Math Methods Med. 2019 Sep 12;2019:3081264. doi: 10.1155/2019/3081264 (PMC6757320; doi:10.1155/2019/3081264)
Supplement: Supplementary Materials — Table S1: age-height percentile values of 0–18 years old children. Table S2: age- body weight percentile values of 0–18 years old children. Table S3: age- body mass index percentile values of 0–18 years old children. [file 3081264.f1.pdf]

TABLE S1: Age-height percentile values of 0-18 years old children.

Age-Height Percentile Values (cm)														
Boy								Girl						
**3**	**10**	**25**	**50**	**75**	**90**	**97**	**Age**	**3**	**10**	**25**	**50**	**75**	**90**	**97**
45.9	47.2	48.5	50.0	51.5	52.9	54.2	**Birth**	45.3	46.6	47.9	49.4	50.8	52.1	53.4
56.2	57.8	59.5	61.3	63.2	64.8	66.4	**3 m.**	55.3	56.8	58.2	59.9	61.5	63.0	64.5
62.8	64.5	66.2	68.0	69.9	71.6	73.2	**6 m.**	61.6	63.1	64.7	66.4	68.2	69.7	71.3
67.4	69.1	70.9	72.8	74.7	76.4	78.1	**9 m.**	66.0	67.7	69.3	71.2	73.0	74.6	76.3
70.8	72.7	74.7	76.9	79.1	81.1	83.0	**12 m.**	69.7	71.4	73.2	75.1	77.1	78.8	80.5
73.8	75.8	77.9	80.2	82.5	84.5	86.6	**15 m.**	72.8	74.6	76.5	78.5	80.6	82.4	84.2
76.4	78.5	80.7	83.1	85.5	87.7	89.8	**18 m.**	75.5	77.4	79.3	81.5	83.7	85.6	87.6
81.0	83.3	85.6	88.2	90.8	93.2	95.5	**2 y.**	80.1	82.3	84.4	86.8	89.2	91.4	93.5
85.3	87.6	90.0	92.6	95.3	97.6	100.0	**2.5 y.**	84.0	86.3	88.6	91.2	93.8	96.1	98.4
89.3	91.7	94.1	96.8	99.4	101.8	104.2	**3 y.**	87.8	90.2	92.7	95.4	98.1	100.6	103.0
92.8	95.2	97.7	100.5	103.2	105.7	108.2	**3.5 y.**	91.1	93.6	96.2	99.0	101.9	104.5	107.0
96.0	98.6	101.1	104.0	106.9	109.5	112.0	**4 y.**	94.3	96.9	99.6	102.5	105.5	108.1	110.7
99.0	101.7	104.3	107.3	110.3	113.0	115.6	**4.5 y.**	97.4	100.1	102.8	105.9	108.9	111.6	114.3
101.8	104.5	107.3	110.4	113.5	116.2	119.0	**5 y.**	100.4	103.2	105.9	109.1	112.2	114.9	117.7
104.5	107.3	110.1	113.3	116.4	119.3	122.1	**5.5 y.**	103.6	106.3	109.0	112.1	115.3	118.3	121.2
107.1	110.0	112.9	116.1	119.3	122.2	125.1	**6 y.**	106.2	109.0	111.9	115.1	118.4	121.3	124.1
112.1	115.1	118.2	121.5	124.9	128.0	131.0	**7 y.**	111.6	114.6	117.7	121.1	124.4	127.5	130.5
116.9	120.0	123.3	126.9	130.5	133.7	136.9	**8 y.**	116.7	119.9	123.1	126.7	130.3	133.5	136.7
121.6	124.9	128.3	132.1	135.9	139.3	142.7	**9 y.**	121.3	124.7	128.2	132.1	136.0	139.5	142.9
126.4	130.0	133.6	137.6	141.6	145.2	148.7	**10 y.**	125.8	129.6	133.5	137.9	142.2	146.1	150.0
131.7	135.5	139.4	143.8	148.1	152.0	155.9	**11 y.**	132.5	136.6	140.8	145.4	150.1	154.2	158.3
137.0	141.3	145.7	150.6	155.4	159.8	164.1	**12 y.**	141.1	144.9	148.8	153.1	157.4	161.2	165.1
142.8	147.6	152.4	157.7	163.1	167.9	172.6	**13 y.**	146.6	150.2	153.8	157.8	161.8	165.5	169.0
150.3	155.0	159.7	164.9	170.1	174.8	179.5	**14 y.**	149.3	152.8	156.4	160.4	164.3	167.9	171.4
156.9	161.2	165.5	170.3	175.1	179.4	183.7	**15 y.**	150.7	154.2	157.8	161.7	165.7	169.3	172.8
160.9	164.9	168.9	173.4	177.9	181.9	185.9	**16 y.**	151.3	154.8	158.4	162.4	166.3	169.9	173.4
163.0	166.8	170.7	175.0	179.3	183.2	187.1	**17 y.**	151.7	155.2	158.8	162.7	166.7	170.3	173.8
164.5	168.2	172.0	176.2	180.4	184.2	187.9	**18 y.**	152.0	155.6	159.1	163.1	167.1	170.7	174.2

TABLE S2: Age- body weight percentile values of 0-18 years old children.

Age-Body Weight Percentile Values (kg)														
			Boy									Girl		
**3**	**10**	**25**	**50**	**75**	**90**	**97**	**Age**	**3**	**10**	**25**	**50**	**75**	**90**	**97**
2.58	2.85	3.13	3.43	3.73	4.00	4.27	**Birth**	2.52	2.76	3.01	3.29	3.58	3.84	4.10
4.75	5.26	5.79	6.38	6.99	7.54	8.10	**3 m.**	4.48	4.90	5.33	5.82	6.32	6.78	7.24
6.21	6.79	7.41	8.12	8.85	9.54	10.25	**6 m.**	5.94	6.38	6.85	7.43	8.06	8.68	9.34
7.27	7.87	8.51	9.26	10.06	10.81	11.58	**9 m.**	6.85	7.34	7.89	8.55	9.29	10.02	10.82
7.96	8.61	9.32	10.16	11.05	11.92	12.82	**12 m.**	7.52	8.06	8.66	9.39	10.20	11.00	11.87
8.61	9.28	10.01	10.89	11.83	12.75	13.72	**15 m.**	8.09	8.67	9.31	10.10	10.96	11.81	12.73
9.13	9.82	10.58	11.49	12.48	13.46	14.49	**18 m.**	8.57	9.19	9.87	10.71	11.63	12.55	13.54
10.12	10.85	11.66	12.66	13.76	14.86	16.05	**2 y.**	9.49	10.20	10.99	11.94	12.99	14.03	15.15
11.06	11.84	12.71	13.80	15.04	16.29	17.69	**2.5 y.**	10.35	11.17	12.06	13.12	14.25	15.33	16.47
11.81	12.65	13.61	14.83	16.24	17.71	19.39	**3 y.**	11.19	12.09	13.05	14.18	15.37	16.51	17.68
12.6	13.5	14.6	15.9	17.4	18.9	20.6	**3.5 y.**	11.9	12.8	13.9	15.1	16.5	17.8	19.3
13.3	14.3	15.4	16.8	18.5	20.1	22.0	**4 y.**	12.7	13.7	14.8	16.1	17.7	19.2	20.8
14.0	15.0	16.2	17.7	19.5	21.3	23.3	**4.5 y.**	13.5	14.5	15.8	17.3	19.0	20.7	22.5
14.7	15.8	17.0	18.6	20.5	22.4	24.6	**5 y.**	14.2	15.4	16.7	18.4	20.3	22.2	24.3
15.4	16.5	17.9	19.6	21.6	23.6	26.0	**5.5 y.**	14.9	16.2	17.7	19.5	21.6	23.7	26.1
16.2	17.4	18.9	20.7	22.8	25.1	27.7	**6 y.**	15.7	17.0	18.6	20.6	22.9	25.3	27.9
18.1	19.5	21.1	23.2	25.8	28.5	31.6	**7 y.**	17.2	18.7	20.6	22.9	25.7	28.6	31.9
19.9	21.5	23.4	25.9	28.9	32.2	36.1	**8 y.**	18.9	20.8	22.9	25.7	28.9	32.4	36.5
21.7	23.6	25.8	28.8	32.4	36.4	41.3	**9 y.**	20.9	23.1	25.6	28.9	32.8	37.0	41.8
23.6	25.9	28.6	32.2	36.7	41.6	47.8	**10 y.**	23.0	25.6	28.7	32.6	37.3	42.3	48.0
26.6	29.6	33.1	37.8	43.6	50.0	57.8	**11 y.**	26.4	29.6	33.4	38.2	43.7	49.5	55.9
29.9	33.8	38.4	44.3	51.3	58.7	67.1	**12 y.**	32.0	35.8	39.9	45.1	50.9	56.8	63.1
33.4	38.0	43.2	49.8	57.3	64.9	73.3	**13 y.**	37.4	41.1	45.1	50.0	55.5	60.8	66.6
39.1	44.0	49.4	56.2	63.9	71.6	80.1	**14 y.**	41.6	45.0	48.8	53.3	58.3	63.2	68.5
45.3	50.1	55.4	62.1	69.7	77.4	85.9	**15 y.**	44.0	47.3	50.9	55.3	60.1	64.8	69.8
49.9	54.5	59.7	66.2	73.6	81.2	89.6	**16 y.**	45.3	48.5	52.0	56.3	61.0	65.7	70.7
53.2	57.8	62.8	69.2	76.5	84.0	92.4	**17 y.**	46.2	49.4	52.9	57.2	61.8	66.4	71.4
56.1	60.5	65.5	71.8	79.0	86.4	94.7	**18 y.**	47.3	50.5	53.9	58.1	62.2	67.3	72.2

TABLE S3: Age- body mass index percentile values of 0-18 years old children.

Age-Body Mass Index Percentile Values (kg/m^2)														
Boy								Girl						
**5**	**15**	**25**	**50**	**75**	**85**	**95**	**Age**	**5**	**15**	**25**	**50**	**75**	**85**	**95**
11.4	12.2	12.7	13.7	14.6	15.2	16.1	**Birth**	11.4	12.2	12.6	13.5	14.4	14.9	15.8
14.4	15.3	15.8	16.9	18.0	18.6	19.7	**3 m.**	13.9	14.8	15.3	16.3	17.3	17.9	18.9
15.0	15.9	16.5	17.5	18.6	19.2	20.3	**6 m.**	14.7	15.4	15.9	16.9	18.0	18.6	19.7
15.1	16.0	16.5	17.5	18.6	19.3	20.4	**9 m.**	14.8	15.5	16.0	17.0	18.0	18.6	19.8
14.9	15.7	16.2	17.2	18.3	18.9	20.0	**12 m.**	14.6	15.3	15.7	16.6	17.7	18.2	19.4
14.7	15.5	16.0	17.0	18.0	18.6	19.7	**15 m.**	14.5	15.1	15.6	16.4	17.4	18.0	19.1
14.5	15.3	15.7	16.7	17.7	18.3	19.3	**18 m.**	14.2	14.9	15.3	16.2	17.1	17.7	18.8
14.3	15.0	15.4	16.3	17.3	17.9	19.0	**2 y.**	14.0	14.6	15.1	15.9	16.9	17.4	18.5
14.2	14.8	15.3	16.2	17.2	17.7	18.8	**2.5 y.**	13.9	14.6	15.0	15.8	16.7	17.3	18.3
13.9	14.6	15.0	15.9	17.0	17.6	18.7	**3 y.**	13.8	14.4	14.8	15.5	16.4	17.0	17.9
13.8	14.5	14.9	15.8	16.8	17.4	18.5	**3.5 y.**	13.7	14.3	14.7	15.5	16.4	17.0	18.0
13.7	14.4	14.8	15.7	16.7	17.3	18.4	**4 y.**	13.6	14.2	14.6	15.4	16.4	17.0	18.1
13.6	14.2	14.7	15.6	16.6	17.2	18.4	**4.5 y.**	13.5	14.2	14.6	15.4	16.5	17.1	18.2
13.5	14.2	14.6	15.5	16.5	17.1	18.3	**5 y.**	13.4	14.1	14.5	15.4	16.5	17.2	18.5
13.4	14.1	14.5	15.4	16.5	17.1	18.4	**5.5 y.**	13.4	14.0	14.5	15.5	16.6	17.3	18.8
13.4	14.1	14.5	15.4	16.5	17.2	18.5	**6 y.**	13.3	14.0	14.5	15.5	16.7	17.5	19.1
13.6	14.3	14.7	15.7	16.9	17.6	19.1	**7 y.**	13.3	14.0	14.5	15.6	16.9	17.8	19.7
13.8	14.5	15.0	16.1	17.4	18.2	19.9	**8 y.**	13.4	14.2	14.7	15.9	17.4	18.4	20.4
14.0	14.8	15.3	16.5	18.0	19.0	21.0	**9 y.**	13.6	14.5	15.1	16.4	18.1	19.2	21.5
14.1	15.1	15.7	17.1	18.9	20.1	22.5	**10 y.**	13.9	14.9	15.6	17.1	19.0	20.2	22.6
14.6	15.8	16.5	18.2	20.4	21.7	24.5	**11 y.**	14.5	15.6	16.4	18.0	20.0	21.3	23.8
15.2	16.5	17.4	19.3	21.7	23.1	26.0	**12 y.**	15.3	16.5	17.3	19.0	21.1	22.3	24.8
15.6	17.0	18.0	19.9	22.3	23.7	26.5	**13 y.**	16.3	17.5	18.3	19.9	21.9	23.1	25.4
16.4	17.7	18.6	20.5	22.8	24.2	27.0	**14 y.**	17.1	18.3	19.0	20.6	22.5	23.6	25.8
17.2	18.5	19.4	21.2	23.4	24.8	27.6	**15 y.**	17.7	18.8	19.5	21.0	22.8	23.9	26.0
18.0	19.3	20.1	21.9	24.1	25.4	28.2	**16 y.**	18.1	19.1	19.8	21.2	23.0	24.0	26.1
18.7	19.9	20.7	22.5	24.7	26.1	28.8	**17 y.**	18.5	19.5	20.1	21.5	23.1	24.2	26.2
19.2	20.5	21.3	23.1	25.2	26.6	29.4	**18 y.**	19.0	19.9	20.5	21.8	23.3	24.3	26.1
